# Beyond Communication and Risk in a Post-Pandemic World: A Survey on Radon in Spain

**DOI:** 10.3390/ijerph22111667

**Published:** 2025-11-03

**Authors:** Jorge Vázquez-Herrero, Berta García-Orosa, Xosé López-García

**Affiliations:** Department of Communication Sciences, Institute of Studies and Development of Galicia (IDEGA), Novos Medios Research Group, Universidade de Santiago de Compostela, Santiago de Compostela, 15782 A Coruña, Spain; berta.garcia@usc.es (B.G.-O.); xose.lopez.garcia@usc.es (X.L.-G.)

**Keywords:** radon, risk communication, risk perception, survey, Spain

## Abstract

This study addresses the construction and perception of risk and the role of the news media through a case study on radon gas, a carcinogenic, persistent hazard with a significant impact on public health, which typically flies under the radar of public opinion. The research is based on a survey (N = 1985) that targeted residents of Spain aged 18 or older. We evaluated cognitive and contextual factors, media consumption, and awareness of radon communication actions and developed a model to explain individual risk perception. The population’s knowledge about the different aspects of the risk of radon gas is moderate and uneven. A key element is the level of radon incidence among Spain’s autonomous communities. The main factors that explain the perception of radon risk are one’s perceived likelihood of being affected by radon, followed by gender and cognitive factors. The results demonstrate a strong correlation between media-disseminated information and public knowledge, but a weaker correlation between such information and protective actions, which are more closely tied to interpersonal and local communication. This study provides insights into addressing new societal risks and will help to create communication tools and analyses that avoid panic and promote responsible actions during crises.

## 1. Introduction

The importance of risk communication, the current state of uncertainty in many aspects of social life, and changes brought about by the pandemic and technological innovations require research on the construction of risks in contemporary societies. Social perception of risk has been studied from various angles [1], but the digital economy creates new risks, involving new actors in public opinion formation and even limiting the scope of democratic decision-making [2].

In this changing context, this study takes individual and social risk perception as a crucial basis for effective communication [3]. Risk-related communication is intrinsic to health, and information on health risks is a necessary component of public health activities [4]. This study deals with risk as a persistent transnational hazard, allowing for analysis beyond specific crises during which roles may temporarily fluctuate due to responsibility or fear [5], or where crises are engendered by specific agents or institutions for their own benefit [6]. We aim to perform a holistic analysis of risk as a social construct based on cognitive elements, social contexts, and emotions, distinguishing between knowledge and perception and their relationship with behavioral change [7].

Through a case study on radon gas and its health implications, we explore trends in risk perception and knowledge, as well as challenges and avenues for effective communication. Radon is the leading cause of lung cancer among non-smokers and the second among smokers, yet few residents of affected areas perceive this risk [8]. At the same time, the media tend to overlook radon, and risk communication efforts to mitigate the effects of this radioactive gas are generally ineffective [9]. Additionally, Council Directive 2013/59/EURATOM mandates that states prepare communication strategies to “increase public awareness and inform local decision-makers, employers, and workers about radon risks”. As such, understanding perception is fundamental for effective risk communication [10] and protecting populations from radon gas.

Our research aims to comprehend the construction and perception of radon risk, as well as the level of knowledge among the Spanish population, due to the high incidence of radon in the country. The radon potential map has established priority action areas that represent 17% of the Spanish territory’s surface area [11,12]. Furthermore, we seek to identify whether sociodemographic variables and radon incidence are determinants in defining risk perception. This objective is a precursor to designing efficient communication actions on radon.

This study addresses a gap in the literature by providing the first nationwide analysis in Spain that integrates individual and social risk perceptions of radon with knowledge levels, preventive behaviors, and exposure to communication channels. While previous research has examined radon’s coverage in the media, few studies have explored how cognitive and emotional dimensions of risk perception interact with information habits and institutional communication. By linking these factors, our work offers empirical evidence to inform targeted risk communication strategies and public health policies.

### 1.1. Risk as a Social Construct

Technically speaking, risk generally refers to an event or the occurrence of an event with a specific and known probability. It is measured in terms of the likelihood of a loss occurring [13] and can be interpreted as the probability that human actions or events will produce harmful consequences for aspects positively valued by society [14]. However, the understanding of risk is socially constructed by individual perceptions and information from others [4], and involves cognitive, contextual, and emotional aspects. Moreover, risk perception consists of shared cognitive schemes that encompass beliefs, attitudes, judgments, and feelings towards hazards and their benefits [15], along with broader social and cultural values [16].

Risk perception is, therefore, a social construct built by different factors and actors that changes significantly across time. Nuclear energy is a classic example, one initially associated with positive images of growth and prosperity. It made its way onto the macropolitical agenda, and its image was greatly tarnished as environmental and health risks emerged [17].

Thus, risks can be hidden, made visible, or even invented. This construction has been analyzed by Kasperson et al. [18] through the Social Amplification of Risk Framework (SARF). This framework describes the processes that can cause some hazards and events, which experts deem to represent a relatively low statistical risk, to become a focus of social attention, concern, and action (amplification) or, conversely, to be minimized (attenuation).

However, the assessment of each actor’s contribution to the construction of that risk has varied. For example, researchers have highlighted the significance of media-disseminated information [19,20] and its importance for public awareness [21], but at the same time, the media are considered sources of risk perception that are prone to dramatization, distortion, sensationalism, misrepresentation, and error. In parallel, researchers have noted that the media construct social but not individual risk perceptions, thus potentially inducing action against radon to a lesser extent [22].

Currently, multiple actors engage in risk communication with different effects. Several studies on social networks and health have been conducted in recent years, highlighting their significance in risk prevention and behavior change [14,23,24,25]. Some studies claim that social networks are more effective than traditional media in disseminating information about health risks [26]. Lee et al. [22] note how active social media users not only share information but also reinterpret risk, indicating how receiving information on social networks correlates positively with personal and social risk perceptions, but not with global risk perceptions.

In our study, risk communication is understood as the management of risk through communication between people, groups, and institutions regarding the assessment, characterization, and prevention or resolution of risk. Communication should be able to persuade the public to change risky attitudes or behaviors, clear up uncertainties as much as possible, and help the community prepare for change [27].

### 1.2. Risk Perception

Although communication can influence risk perception, there are several additional factors to consider. Other risk communication scholars have found that risk acceptability was determined by two key components: hazard and outrage [28]. Krause et al. [29] echo this by stating that hazards characterized by a greater number of outrage factors tend to provoke emotional reactions that can increase risk perception. The public often perceives risk as something that directly affects them [30]. Other identified factors include potential catastrophe, familiarity, difficulty in understanding, level of scientific uncertainty, controllability, voluntariness, trust in institutions, and media attention.

In addition to these intrinsic components of risk and its context, communication between the source and the audience plays a fundamental role in risk perception. Currently, traditional problems persist [31], among which are limitations in the scientific assessment of risk; relationships with information sources and channels; and messages focused on increasing awareness of the risk that do not address behavioral barriers and recommendations [9].

### 1.3. Radon Gas and How It Is Perceived

Radon is a radioactive gas that is odorless, tasteless, and colorless. Recognized as a carcinogen by the World Health Organization in 1988 [32], it is the leading cause of lung cancer among non-smokers and the second biggest cause among smokers [33]. Its significance lies in its being a persistent risk, with serious health effects demonstrated for nearly 40 years. Nonetheless, despite the severity of the risk radon presents, there is limited public awareness thereof [34]. Anywhere from 3% to 14% of lung cancer cases can be attributed to radon [8], depending on a country’s average radon concentration and the calculation method used.

Recent studies have indicated that radon gas has intrinsic elements associated with a reduced risk perception, as it is currently communicated as a ‘natural‘ risk that neither poses an imminent danger nor is the fault of any specific party [9]. Framing it as a ‘natural‘ risk complicates communication by causing radon to be seen as a chronic rather than acute risk, without an obvious responsible party or social or political conflict—thus without the outrage factor discussed by Malecki et al. [28]. Additionally, because radon must be measured to be detected, it can go unnoticed [30] despite its severe effects. Lastly, the cost of mitigation for homeowners, builders, and real estate agents can be significant.

Risk perception has been measured in recent years through surveys [35,36,37,38,39]. A radon survey on buildings in 23 European countries by Holmgren et al. [40] emphasized the lack of prevention and the need for effective communication. In Russia, Davydov et al. [41] suggested that understanding the similarities between health-related knowledge, attitudes, and behaviors and those related to radon may require effective radon-related education strategies in specific populations. They delineated a series of stages for a population’s perception of radon: ignorance about radon protection, awareness but no engagement, engagement and decision-making about specific actions, decision to take no action, decision to take specific actions but no follow-through, action (occasional and/or periodic), and consolidation of a protective health practice.

Our study is part of a line of research that has emerged since the COVID-19 pandemic, wherein researchers are seeking to identify risk prevention tendencies. Dryhurst et al. [20] correlate risk perception with the adaptation of health behaviors and highlight, among several influencing factors, personal experience as well as trust in information sources and access to communication strategies.

The systematic review on radon risk perception by Cori et al. [42] points to growing scientific interest and identifies risk perception and communication as key topics, citing several articles from different countries, but none focusing on Spain, where the incidence of radon is particularly high in some regions. Based on previous research [43,44,45] and the reality of radon as a natural risk, we analyze risk perception from a perspective that includes the probability of people and the immediate environment being affected, with a specific focus on the level of knowledge from communication actions and measures, and the influence of media, in addition to sociodemographic variables and incidence in each of Spain’s autonomous communities.

In summary, we approach risk as a social construct that is influenced by various factors and communicated through traditional, digital media, and social networks, considering that risk perception is shaped in the communication process and can have significant implications on people’s behavior and attitudes.

## 2. Materials and Methods

**Figure 1:**
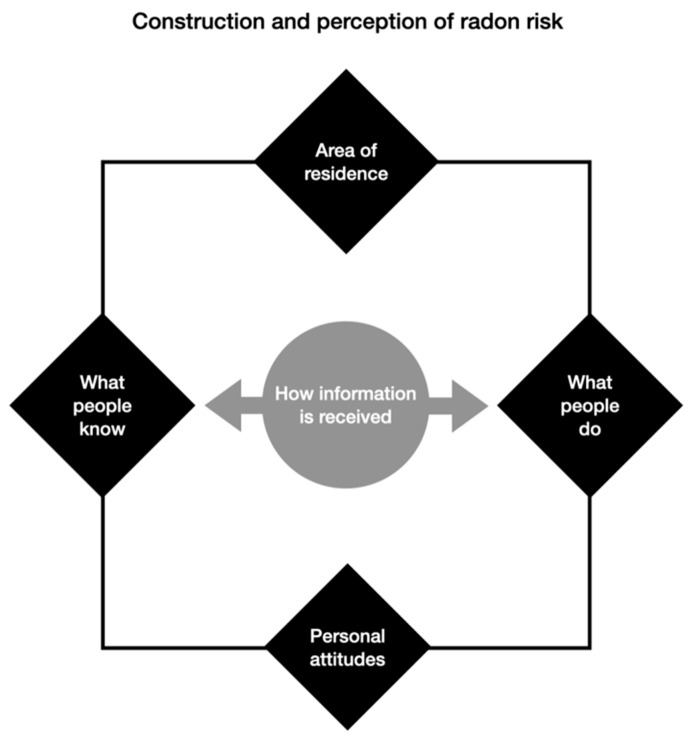
Base model for the study (created by authors).

Our study begins with the question: “What is the population’s concept of radon risk and how is it constructed?” It is oriented around the following initial hypotheses, derived from a review of the literature on risk perception in the case of radon and other phenomena related to public health. Considering place of residence as a relevant factor—given radon’s varying incidence by location—as well as personal attitudes, this study explores the construction and perception of risk based on what citizens know about radon and what they do to prevent and mitigate the risks thereof and protect themselves, from a communication perspective. This means examining how information influences their knowledge and actions, which in turn impacts risk perception (Figure 1). As such, we propose the following hypotheses:

**H1.**  *People in areas more affected by radon exhibit slightly higher risk perception.*

**H2.**  *Individuals who report receiving information about radon through media channels tend to show higher levels of risk perception.*

**H3.**  *Levels of knowledge and information are associated with greater awareness of risk and the adoption of preventive or mitigating actions.*

This study employs a survey to understand public perception of radon risk. This technique has been validated in previous studies on various risks such as climate change, radon gas, Ebola, and COVID-19 [20,40,46,47]. The method employs a multi-perspective approach to analyze risk communication from different viewpoints. The survey, comprising 41 questions across seven blocks, covers areas such as social and individual perception of radon risk, knowledge about radon, awareness of radon events, understanding of political and legislative actions regarding radon, and personal actions taken against radon. Demographic variables recorded include age, gender, place of residence, education level, employment status, occupation, and income level.

Conducted online using the CAWI system, the survey targeted residents of Spain aged 18 or older. Data collection took place from 1 March to 5 June 2023. A multi-stage sampling method was used, with territorial quotas to proportionally allocate 1500 respondents (by autonomous community and place of residence), gender, and age, plus random household selection. An additional oversampling of up to 300 responses was performed in the most affected territories for deeper local analysis. The maximum admitted error for the entire sample is ±2.18%, with a confidence level of 95.5%, and *p* = Q as the most unfavorable case. The total valid sample (Table 1) was N_1_ = 1985 respondents, including oversamples in the most affected territories, and N_2_ = 1442 for proportional national representation.

For the analysis, IBM’s SPSS Statistics 28 was used, applying descriptive statistics to analyze the variables and their grouping into dimensions. The internal consistency of these dimensions was tested using Cronbach’s alpha test. The analysis included:

- Individual and social perception of radon risk: Assessed via a series of nine Likert scale (1–7) questions, based on Morton and Duck’s [48] approach to measuring personal risk perception, adapted for assessing risk perception in others and nearby environments. It included a set of questions for “Individual Risk Perception” (14 variables, α = 0.924) and “Social Risk Perception” (10 variables, α = 0.842).
- Knowledge about radon: Evaluated through seven dichotomous response questions, forming a “Knowledge” dimension (20 variables, α = 0.824) representing the degree of knowledge about this gas.
- Knowledge of radon events: Assessed with two dichotomous response questions, forming an “Events” dimension (3 variables, α = 0.658).
- Knowledge of political and legislative actions on radon: Assessed with eight dichotomous response questions, forming a “Political and Legislative Actions” dimension (6 variables, α = 0.825).
- Preventive, mitigative, and protective measures: Evaluated through three dichotomous or multiple-choice response questions, forming a “Measures” dimension (11 variables, α = 0.716).
- Information habits and communication actions on radon: Assessed via Likert scale (1–7), multiple-choice, and dichotomous response questions.

The survey instrument, including questions, variables, and scoring ranges, is available as Supplementary Material File S1. The statistical analysis included calculating variable correlations and an analysis of variance (ANOVA) according to sociodemographic variables. A linear regression model was used to predict individual risk perception, yielding an R-squared of 0.661, indicating a good result, taking into account that it predicts a social behavior.

## 3. Results

### 3.1. Cognitive and Contextual Factors

**Figure 2:**
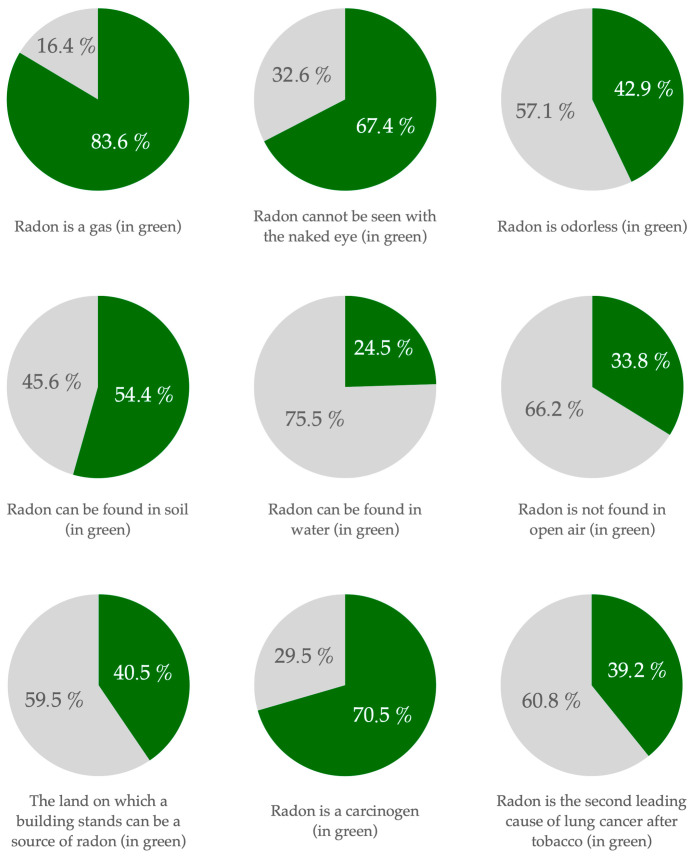
Graphs based on radon awareness (created by the authors based on the survey).

The Spanish population demonstrates moderate knowledge about radon (Table 2, Figure 2): 83.4% correctly identify it as a gas, 67.4% know it is invisible to the naked eye, and 42.9% understand it is odorless. In total, 54.4% and 24.5% are aware that radon can be found in soil and water, respectively, but only 33.8% know that it is not found in open air. About 40.5% are aware that the ground a building sits on can be a source of radon. Radon is recognized as a carcinogen by 70.5%, but only 39.2% know it is the second leading cause of lung cancer after tobacco. Knowledge about measures to reduce its effects is lower: 75.4% mistakenly believe avoiding living near polluting factories can reduce risks, while effective measures like ventilating closed spaces (39.1%) and ventilating underfloor areas (35.6%) are less known. The results show a higher level of awareness in communities with high radon incidence (*F* = 6.129; *p* = 0.013), especially when it comes to identifying unrelated factors and knowing radon’s characteristics and consequences. For example, 77.0% of participants in the most affected communities recognize radon as a carcinogen, while 19.7% do not know or do not answer. In the rest of the communities, the results reach 68.8% and 26.0%, respectively. When it comes to gender, men show a higher level of knowledge about radon, as well as respondents with higher education levels (bachelor’s, master’s, and doctorate) (*F* = 7.137; *p* < 0.001).

For radon-related events, knowledge is even lower, with about 33% aware of EU sanctions against Spain for delaying radon regulations, high natural radioactive gas concentrations in the Madrid mountain range detected in 2019, or high radon levels in Galician secondary schools. Younger age groups (18–29 and 30–44) have more knowledge about these events (*F* = 14.757; *p* < 0.001), as well as more educated respondents (*F* = 4.586; *p* < 0.001). On the other hand, no significant differences were found based on factors such as community incidence, possibly due to the specific nature of this information and its generally low impact. There is also a limited perception of receiving information through media and social networks.

Regarding political–legislative actions, there is a significant lack of knowledge, since all the questions received “don’t know” as the majority answer. Here, we refer to regulations, recommendations, and radon measurement in living environments, where only between 11% and 39% of the respondents indicated that they were aware of these issues. No significant differences were identified in communities with a high incidence, but there were significant differences by age—the younger the people, the greater the knowledge, although it was also limited.

### 3.2. Media Consumption and Radon Communication Actions

Respondents express a moderate to high interest in staying informed about their surroundings and the world (5.42 out of 7), with 21.9% considering themselves extremely interested and 2.9% showing no interest in current affairs; 77.0% indicate interest (responding from 5 to 7 on a 1–7 scale). Regarding news avoidance, 27.5% actively do it (responding from 5 to 7 on a 1–7 scale). A small percentage (7.9%) shows the highest level of interest in staying informed while also avoiding news, suggesting a broader understanding of “staying informed” beyond traditional news and media, for example, by staying up to date on their friends or family members or their communities, another aspect that should be taken into account.

Approximately 31.2% of citizens report having received information about radon through media at some point—via television (30.2%), digital media (20.1%), print media (12.9%), or radio (11.5%)—with higher rates in communities with greater radon incidence (*F* = 3.384, *p* = 0.066) and in the 30–44 age group (*F* = 2.326, *p* < 0.073). This correlates with knowledge about radon, related events, and political actions, where information received through media positively affects knowledge levels (*p* < 0.001), doubling the score of knowledge about actions.

Regarding communication actions, citizens’ perception of being informed by institutions or groups is low (Table 3). The media ranks highest in this regard (19.5%), followed by the European Union (10.3%) and scientific bodies (8.4%). These findings are predominantly correlated with the level of knowledge about radon, events, actions, and measures; moreover, the strongest correlation observed with the radon knowledge indicator is with the ‘media’, underscoring the importance of this channel in disseminating general information.

### 3.3. Prevention, Mitigation, and Protection

The population has taken a few measures to protect against radon, with only 45.6% adopting some form of protection. The most common measures are improving building ventilation (29.9%), followed by increasing underfloor ventilation (21.7%), preventing gas infiltration from the basement (15.6%), sealing floors and walls (13.0%), and installing a mechanical radon evacuation system in the basement or under solid floors (10.5%).

Knowledge about protective measures is very low, with a majority unaware of how to conduct a radon test, where to obtain a test kit, or how to find an experienced contractor for home remediation.

The indicator on prevention, mitigation, and protection measures correlates positively (*p* < 0.001) with knowledge about radon, related events, and political–legislative actions (H3). Measures are more commonly taken among those informed by the media (*p* < 0.001), attaining a 4.25 out of 7 score (SD = 1.950), compared to a score of 3.09 (SD = 1.551) among those who stated that they have not received information about radon from the media. The most pronounced difference (*p* < 0.001) is identified between those who have been informed about radon through social media (4.97; SD = 1.995) and those who did not receive information on such platforms (3.31; SD = 1.647). Unlike the radon knowledge indicator, which has a strong correlation with the media as the source of communication actions, the strongest correlations for the “taking measure” indicator are with ‘workplace’, ‘school’, and ‘college’, indicating effective communication spaces for prevention, mitigation, and protection.

### 3.4. Risk Perception of Radon

The Spanish population generally feels more concerned about issues in general (4.68 out of 7) than about the risk associated with radon gas (4.04), with greater values found in autonomous communities that have higher radon incidence, especially in Galicia (4.58) but also in Madrid (4.19) and Extremadura (4.09), although the explained variance is minimal in the ANOVA test (H1). One-way ANOVAs indicated statistically significant group differences for ‘Worried about radon affecting me today’ (F = 5.360, *p* = 0.021), ‘Likelihood (personal)’ (F = 9.083, *p* = 0.003), and ‘Likelihood (others)’ (F = 8.806, *p* = 0.003). Means (95% CIs) for the low-incidence versus high-incidence regions were, respectively, 3.79 [3.69, 3.89] vs. 3.97 [3.85, 4.09] for ‘Worried about radon affecting me today’, 3.40 [3.30, 3.50] vs. 3.62 [3.51, 3.73] for ‘Likelihood (personal)’, and 3.28 [3.18, 3.38] vs. 3.50 [3.39, 3.61] for ‘Likelihood (others)’. The mean differences (high-low) were 0.18 [0.02, 0.34], 0.22 [0.07, 0.37], and 0.22 [0.08, 0.36], respectively. Consistent with the ANOVA effect-size estimates, these differences were very small (ω^2^ ≈ 0.002–0.004; all 95% CIs included 0), indicating minimal variance explained despite statistical significance.

From highest to lowest, the phenomena and diseases whose risk generates the greatest concern are: an economic crisis, climate change, a fire, a pandemic, a nuclear accident, or an earthquake. However, respondents’ perceived probability of being affected by radon today is lower (3.44), which translates into moderate concern about radon affecting them today (3.81). The perceived probability of radon affecting friends or family is also moderate (3.28). These measurements of individual risk perception correlate with each other, as well as with the level of knowledge about radon, events, and actions (H3), and with the consumption of media-disseminated information about radon (*p* < 0.001 for each variable), with higher results when respondents report having received information through the media (H2). Likewise, variables such as the probability of being affected by radon (*F* = 2.269, *p* < 0.002) or of radon affecting their surroundings (*F* = 2.109, *p* < 0.004) vary significantly according to the community, so much so that those more affected show higher results; this is also true for women (*p* < 0.001).

Having evaluated the existing correlations, a linear regression model was built to explain individual risk perception, taking the question “To what extent are you concerned that radon will affect you today?” as a dependent variable (affective), to best fit the model. In the first block of a hierarchical regression (model 1), we included only cognitive predictors as independent variables, which significantly influence the dependent variable: knowledge about radon, knowledge of political–legislative actions, perceived probability of being affected today, perceived probability of friends and/or family being affected today. This model explained 53.7% of the variance in individual risk perception (R^2^ = 0.537, *p* < 0.001). In the second block (model 2), we added demographic variables (region with high radon incidence, gender, and age) as independent variables. The change in explained variance was minimal (ΔR^2^ = 0.002, *p* = 0.020), indicating that cognitive factors are the primary drivers of individual concern.

The model yielded the following results (Table 4) with significant ANOVA, Durbin–Watson in normal values, VIF below 10, R-squared of 0.539, reflecting a good outcome considering that it predicts a social behavior. Thirty-five cases were determined to be outliers and were removed.

**Figure 3:**
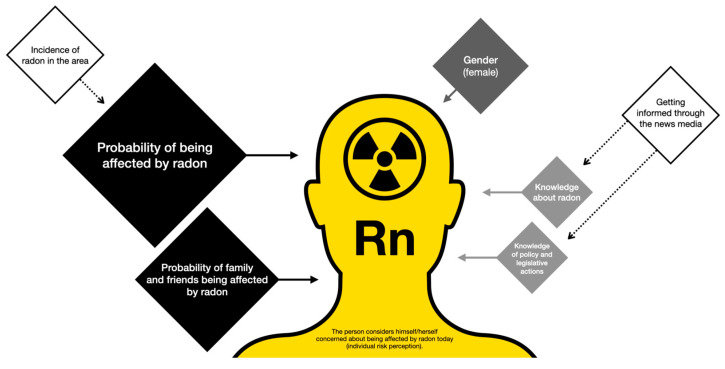
Visual representation of the factors identified in the individual perception of risk (created by authors based on the model in Table 4).

This indicates that the individual risk perception (Figure 3), based on the item “To what extent are you concerned about radon affecting you today?” is constructed on the basis of the perception of the probability of being affected and of family and friends being affected, followed by gender and—with lower influence—knowledge about radon and about the political–legislative actions taken.

In terms of social perception of risk, the perception of incidence in the community in which one resides (3.34 at the national level) presents higher results (with a statistically significant difference) in regions with higher incidence (H1; *F* = 71.985, *p* < 0.001), with the highest score in Galicia (4.32), the most affected community. Similarly, with statistically significant differences, the results are also higher among women (*F* = 5.954, *p* < 0.005). Regarding associated risks, respondents rank the risk to public health highest (5.22), again with higher scores in the most affected communities (*F* = 7.693, *p* < 0.01) and women (*p* < 0.001), while the lowest scores are found among respondents with the lowest levels of education.

We also identified multiple correlations between variables of this dimension and the level of knowledge about radon, associated events, and political–legislative actions. Specifically, identifying radon as a serious health risk correlates with respondents’ perception of being affected themselves, with their perception of their friends and family being affected, and with their level of knowledge about radon, events, and actions, thus highlighting the importance of risk communication. Moreover, the values for this item are higher in communities with high incidence (Galicia has the highest levels) and among women.

Among the social perception of risk variables, the lack of knowledge about whether the home or workplace has radon problems stands out (65.8% rate between 5 and 7). Also noteworthy is the low perception of having received information about the radon situation in their community or country (15.9% rate from 5 to 7), in this case with a statistically significant difference and a slightly higher perception in the most affected communities (*F* = 8.251, *p* < 0.01).

For social perception of risk, it was not possible to form a model, but correlations were identified between multiple variables that indicate a close and significant relationship between information habits and communication actions about radon and the social perception of risk. Therefore, the fact of having received information about radon through the media, having been informed by institutions or groups, as well as knowing that specific communication activities about radon have been carried out in the country is related to the perception of the incidence of radon in the community in which they live, as well as the risk to public health, among other variables. However, without being able to affirm that a low level of information causes a lower perception of risk, these correlations suggest that information in the media and institutional communication actions are key to building social perception of radon-associated risks. We can also assert that concern about what happens to friends and family correlates positively with social perception of risk.

Comparing the correlations between individual and social risk perception with the scores for prevention, mitigation, and protection measures, we observe that taking such measures correlates more strongly with individual rather than social perception of risk. Specifically, the strongest correlation occurs with the concern of being affected today by radon (r = 0.210; *p* < 0.001). This indicates that citizens take more measures when they perceive that radon affects them personally, rather than their surroundings or other people.

## 4. Discussion and Conclusions

Radon risk lies in a complex dimension in which cognitive and emotional components are deeply intertwined [42], which helps explain why knowledge alone does not always translate into preventive action. The survey revealed moderate and uneven knowledge about radon among the Spanish population. While most respondents can correctly identify radon as a gas (83.4%) and recognize some of its characteristics, awareness of its health risks and effective mitigation measures is limited. Knowledge is higher in high-incidence regions. Awareness of radon-related events and political–legislative actions is notably low, with most respondents unable to identify regulations or sanctions related to radon. Media exposure to radon information is scarce (31.2%) but positively associated with knowledge and preventive behaviors. Overall, only 45.6% report adopting any protective measures, mainly ventilation improvements. Risk perception is moderate, slightly higher in high-incidence areas, and correlates with knowledge and media exposure, although the explained variance is minimal.

Previous studies have analyzed radon and its portrayal in the Spanish media [49,50,51]; thus, this study’s survey addressed the perception of radon risk in Spain and how it has arisen as a social construct. Although the study has a national focus, it can be interpreted in global terms, taking into account the levels of affectation in different territories. Although there may be social, cultural, economic, or political specificities, the patterns observed may have implications beyond Spain, although further research is needed to confirm their applicability in other contexts.

The population’s knowledge about the different aspects of the risk of radon gas is moderate and uneven, revolving mostly around radon’s characteristics rather than its health effects or mitigation measures, as also found in previous studies on the media agenda. Following Davydov et al. [41], we can indicate that the Spanish population falls between the first two phases of their process: “ignorance about radon protection” and “awareness but no engagement”. However, it has been shown that cognitive, contextual, or communicative factors significantly influence risk perception: generally, having knowledge results in risk perception and a predisposition to act against radon.

A key element is the level of radon incidence in Spain’s autonomous communities. In the most affected areas (H1)—Galicia, Extremadura, and Madrid—a significant difference was identified with greater knowledge about radon’s characteristics and consequences, information received about the gas through media, concern about radon, likelihood of being affected, and social perception of risk—including perception of radon-associated risk. However, community radon incidence does not significantly affect knowledge about events and political–legislative actions. This may reflect the limited dissemination of such information, as suggested by the low reported exposure in the survey.

We employed a linear regression model to explain individual perception of radon risk. The main factors are one’s perceived likelihood of being affected by radon, and also affecting family and friends, followed by gender (female) and—with lower impact—knowledge about radon and about the political–legislative actions taken. Nonetheless, respondents’ concerns about radon-related risks were greater than their perception of how likely they are to be affected personally. Prior studies have also found that people perceive radon to represent an attenuated risk because they perceive the gas to be something “natural” [9]. Both variables are higher in the most affected communities, making location-based risk communication fundamental. Similarly, the media are frequent channels for this purpose, and individual risk perception partially stems from knowledge about radon and political–legislative actions.

Through a more limited correlation analysis in the social perception of risk, radon incidence in one’s community, and radon knowledge arise as significant variables. A significant relationship has also been identified between consumption of media-disseminated information (H2) and communication actions with social risk perception, thereby highlighting how media and institutions play a key role in social risk communication.

Our analysis has identified that people perceive that they have received little media-disseminated information, but that this information has a positive influence on several variables. Respondents who report having received media-disseminated information about radon also indicated greater knowledge about radon, related events, political–legislative actions, and measures. Observing the origin of communication actions, we identify a strong correlation between media-disseminated information and knowledge about radon. In line with Lee et al. [22], the media contributes to social perception and, to a lesser extent, may induce people to act, as do labor and educational institutions, which leads to a greater disposition to taking prevention, mitigation, and protection measures, indicating the most efficient channels for specific messages in risk communication.

Previous studies have correlated risk perception with the adoption of health behaviors and highlighted factors such as personal experience, information sources, and access to communication strategies [20]. In this study, risk perception increases the adoption of prevention, mitigation, and protection measures when it comes to individual or social perception, but individual perception is a stronger influence than social perception, revealing that citizens take more measures when they perceive that radon affects them directly. Thus, taking action is linked more to contextual or local and interpersonal communication aspects than cognitive ones. In this way, people in Spain’s most affected autonomous communities are more aware of radon and its effects, and information received from local institutions or one’s close personal relationships has a positive effect on action. In accordance with hypothesis H3, one’s level of knowledge and information implies awareness as well as taking action in terms of prevention, mitigation, and protection. Moreover, having received information either through the media or social networks leads to greater adoption of such measures.

We confirm, following other studies [22], the trend towards the social construction of risk (i.e., a perception that risks affect other people or society in general) based on community radon incidence, information in the media, and radon-related communication actions. In contrast, individual perception is more related to how likely one perceives that they will be affected by radon and the impact it may have on their family and friends.

This study has some limitations, although it has addressed radon at a nationwide level using a method that ensures representation of different regions. One aspect that demands more attention is the flow of communication through social networks: although these platforms are a fundamental channel for accessing information, only 19.4% of respondents indicated that they had received information about radon on social networks. This finding is consistent with evidence reported in studies conducted in other countries [14]. Moreover, given today’s digital media ecosystem, more research is needed on risk communication on social networks and public engagement.

In conclusion, despite radon being a serious, persistent risk with a significant impact on public health, the Spanish population shows only moderate awareness of its incidence and consequences, especially in the most affected communities, highlighting the importance of targeted communication strategies for specific populations [40,41]. Information in the media and local institutional communication actions are recommended as critical for fostering risk perception so as to lead people to adopt conscientious behaviors and take action.

## Figures and Tables

**Table 1:** Breakdown of the sample (N_1_ = 1985).

Age (Years)	%
18–29	13.6
30–44	31.4
45–64	48.3
≥65	6.7
Gender	%
Female	51.2
Male	48.7
Other	0.1
Education level	%
No formal education	0.7
Primary school	1.9
Secondary school	26.9
Professional training	27.6
Bachelor’s Degree	19.5
Master’s Degree	18.9
PhD	4.4
Employment status	%
Student	4.7
Paid full-time job	56.2
Paid part-time job	11.6
Unpaid work (e.g., household work)	4.6
Unemployed	13.3
Retired or pensioner	9.6
Monthly income (euros)	%
No income	7.2
<500	5.8
500–1000	13.5
1000–1500	23.1
1500–2000	17.6
2000–2500	12.9
2500–3000	8.8
3000–5000	7.6
5000–7000	1.6
7000–9000	0.9
≥9000	1.1

**Table 2:** Levels of knowledge about radon, events, and political–legislative actions, indicating the variables that represent statistically significant differences.

	High Incidence Area	Other Areas	Gender	Age	Formal Education
Knowledge of radon	M = 10.80	M = 9.85	+M	–	+University studies
Knowledge of radon events	M = 1.10	M = 1.00	+M	–	+University studies
Knowledge of radon-related political and legislative actions	M = 0.97	M = 0.93	+M	+18–29/ 30–44	–

**Table 3:** Respondents who have received information from institutions or organizations.

Institution/Organization	Respondents (%)
School	4.8
University	3.5
Workplace	8.0
Media	19.5
National government	7.6
Regional government	6.2
Municipal government	4.6
European Union	10.3
Scientific organizations	8.4
Nuclear Safety Council of Spain	4.6
Friends and family	8.0
Others	0.1
None	58.2
No response	3.1

**Table 4:** Linear regression model for individual perception of radon risk.

Model	R	R^2^	Adjusted R^2^	Std. Error	Change Stats						Durbin–Watson	
					Change R^2^	Change F		gl1	gl2	Sig. Change F		
1	0.733	0.537	0.537	1.218	0.537	573.162		4	1979	0.000		
2	0.734	0.540	0.539	1.216	0.003	3.741		3	1976	0.011	1.824	
Coefficients ^a^												
Model			Non Std. Coeff.		Std. Coeff.	t	Sig.	Correlations			Colinearity Stats	
			B	Dev. Error	Beta			Zero order	Partial	Part	Tolerance	VIF
1	(Constant)		0.741	0.087		8.474	<0.001					
	Probability of being affected by radon		0.465	0.025	0.442	18.963	<0.001	0.694	0.392	0.290	0.431	2.321
	Probability of family and friends being affected by radon		0.346	0.025	0.442	13.758	<0.001	0.665	0.295	0.210	0.431	2.320
	Knowledge about radon		0.029	0.007	0.072	4.509	<0.001	0.124	0.101	0.069	0.921	1.086
	Knowledge of policy and legislative actions		0.048	0.019	0.040	2.481	0.013	0.197	0.056	0.038	0.890	1.123
2	(Constant)		0.549	0.136		4.035	<0.001					
	Probability of being affected by radon		0.462	0.025	0.439	18.873	<0.001	0.694	0.391	0.288	0.430	2.325
	Probability of family and friends being affected by radon		0.343	0.025	0.318	13.668	<0.001	0.665	0.294	0.209	0.430	2.325
	Knowledge about radon		0.031	0.007	0.076	4.709	<0.001	0.124	0.105	0.072	0.904	1.106
	Knowledge of policy and legislative actions		0.053	0.020	0.044	2.676	0.008	0.197	0.060	0.041	0.860	1.163
	Region (high radon incidence)		−0.005	0.055	−0.001	-0.097	0.923	0.053	−0.002	−0.001	0.990	1.010
	Gender (female)		0.180	0.055	0.050	3.247	0.001	0.109	0.073	0.050	0.973	1.028
	Age		0.002	0.002	0.017	1.065	0.287	−0.039	0.024	0.016	0.955	1.047

^a^ Dependent variable: P19 “To what extent are you concerned about radon affecting you today?”

## Data Availability

Data in this study will be shared upon reasonable request to the corresponding author and by following the data management guidelines of this project.

## Supplementary Materials

The following supporting information can be downloaded at: https://www.mdpi.com/article/10.3390/ijerph22111667/s1, File S1: Survey on radon risk perception and communication.
