# Supplementary material for: Beyond Communication and Risk in a Post-Pandemic World: A Survey on Radon in Spain
Source: Int J Environ Res Public Health. 2025 Nov 3;22(11):1667. doi: 10.3390/ijerph22111667 (PMC12652366; doi:10.3390/ijerph22111667)
Supplement: Supplementary file 1 [file ijerph-22-01667-s001.zip › ijerph-3871988-supplementary.pdf]

List of questions and variables

- **Individual and social perception of radon risk**: Assessed via a series of nine Likert scale (1–7) questions, based on Morton and Duck's approach to measuring personal risk perception, adapted for assessing risk perception in others and nearby environments. It included a set of questions for "Individual Risk Perception" (14 variables, α = .924) and "Social Risk Perception" (10 variables, α = .842).

Individual risk perception

Question	Variables	Answer
17a. In general, do you consider yourself a worried person?	17a Worried person	1 (Not worried at all) - 7 (Extremely worried)
17b. Are you worried about the risk associated with radon gas?	17b Worried about radon risk	
18. Are you worried about the risk associated with the following phenomena and diseases?	18_1 Climate change	
	18_2 Economic crisis	
	18_3 Earthquake	
	18_4 Fire	
	18_5 Monkeypox	
	18_6 COVID-19	
	18_7 Another pandemic	
	18_8 Radon exposure	
	18_9 Nuclear accident	
19. To what extent are you worried that radon may affect you today?	19 Worried about radon affecting today	
20. What do you think is the likelihood that radon may affect you today?	20 Likelihood (personal)	
21. What do you think is the likelihood that your friends or family are affected by radon today?	21 Likelihood (others)	1 (Very low) - 7 (Very high)

Social risk perception

Question	Variables	Answer
22. What do you think is the incidence of radon in the autonomous community where you live?	22 Incidence perception	1 (Very low) - 7 (Very high)
23. To what extent do you agree with the following statements?	23_1 Radon does not affect many people or homes in my autonomous community	1 (Strongly disagree) - 7 (Strongly agree)
	23_2 Radon does not affect many people or homes in Spain	
	23_3 My autonomous community is the area in Spain with the most municipalities affected by high radon concentrations	

	23_4 Madrid is the region in Spain with the most municipalities affected by high radon concentrations	
24. To what extent do you agree with the following statements?	24_1 Radon is an environmental risk	1 (Strongly disagree) - 7 (Strongly agree)
	24_2 Radon is a public health risk	
	24_3 Radon is an economic risk	
	24_4 Radon is not a risk	
25. To what extent do you agree with the following statements?	25_1 I have received information about the radon situation in my autonomous community/country	1 (Strongly disagree) - 7 (Strongly agree)
	25_2 I do not know whether my home or workplace has a radon problem	
	25_3 I am aware that my home or workplace has high radon levels	
	25_4 Radon is a serious health risk	
	25_5 Radon can be controlled through measurements and ventilation	

Note: Variables 25_1, 25_2, 25_3 and 25_5 excluded in the "Social risk perception" dimension (not related).

- **Knowledge about radon**: Evaluated through seven dichotomous response questions, forming a "Knowledge" dimension (20 variables, α = .824) representing the degree of knowledge about this gas.

Question	Variables	Answer
1. Regarding radon, indicate whether the following statements are true or false	1_1 Radon is a gas	True; False; Don't know/No answer
	1_2 Radon cannot be seen with the naked eye	
	1_3 Radon has no smell	
	1_4 Radon has a strong smell – *Control item*	
2. Where can radon be found? Indicate whether the following statements are true or false	2_1 In open outdoor spaces	True; False; Don't know/No answer
	2_2 In the ground	
	2_3 In water	
3. Indicate the possible sources of radon in the home or workplace	3_1 The ground on which the building stands	Multiple choice
	~~3_2 Industrial pollution~~	
	3_3 Household appliances	
	3_4 Construction materials	
	~~3_5 Domestic water supply~~	
	3_6 Don't know	
4. Exposure to high concentrations of radon in the air can cause lung cancer	4 Association with lung cancer	True; False; Don't know/No answer
5. Of the following statements about radon, indicate which one is true	Radon is a chemical that appears in the atmosphere as a result of industrial processes and is therefore a common pollutant.	Single choice
	Radon is a gas that occurs naturally in soil, water, and rocks as a result of the radioactive decay of other radioactive isotopes.	
	Radon is the result of scientific experiments and the industrial use of nuclear energy.	
	Radon is a natural product that can be used for energy production and has industrial applications.	

	None are true.	
	All are correct.	
6. Indicate which of the following statements are true or false	6_1 Radon is the second leading cause of lung cancer, right after tobacco	True; False; Don't know/No answer
	6_2 Radon is an essential raw material for X-rays and radiotherapy treatments in hospitals	
	6_3 Radon is an essential element in many medical treatments	
	6_4 The presence of radon in enclosed spaces directly causes various types of cancer and other diseases	
7. Mark with an X the measures you consider relevant to reduce radon levels in buildings	7_1 In many cases, radon levels can be reduced by ventilating	Multiple choice
	~~7_2 Radon levels can be reduced by avoiding living near polluting factories~~	
	7_3 I am unaware of radon reduction systems	
	7_4 Radon can be reduced through construction solutions, such as ventilating the crawl space	
	7_5 None	
	7_6 Don't know	

Note: Struck-through variables were excluded for being outliers in the "Knowledge" dimension.

- **Knowledge of radon events**: Assessed with two dichotomous response questions, forming an "Events" dimension (3 variables, α = .658).

Question	Variables	Answer
8. Indicate which of the following statements are true or false	8_1 The EU opened a sanctioning procedure against Spain due to delays in approving regulations on radon gas	True; False; Don't know/No answer
	8_2 In 2019, high concentrations of a naturally occurring radioactive gas were discovered in the Sierra de Madrid	
	8_3 A study found high concentrations of radon in Galician high schools	

Note: An additional question asked "9. Are you aware of the existence of a European Radon Gas Day? Yes (please indicate the date); No".

- **Knowledge of political and legislative actions on radon**: Assessed with eight dichotomous response questions, forming a "Political and Legislative Actions" dimension (6 variables, α = .825).

Question	Variables	Answer
34. Is there any activity related to radon and/or any regulation or guidance material at the regional or national level that addresses radon in housing?	34 Actions addessing radon in housing	Yes; No; Don't know
35. If you are going to rent or buy a home, should radon be measured?	~~35 Radon measure renting a house~~	
36. Is radon measurement in newly built homes a mandatory requirement in Spain?	36 Radon measure in new houses	

37. Are there regulations or guidelines on radon mitigation and prevention in buildings in Spain?	37 Regulations or guidelines in building	
38. Are there regulations or guidelines on radon mitigation and prevention in workplaces in Spain?	38 Regulations or guidelines in workplaces	
39. Are there regulations or guidelines on radon mitigation and prevention in leisure spaces in Spain?	39 Regulations or guidelines in leasure spaces	
40. Are you aware that in December 2022 a new regulation on radon was approved in Spain, which includes the launch of a National Radon Plan?	40 National Radon Plan	
41. Do you know of any study on radon?	~~41 Studies~~	

Note: Struck-through variables were excluded for being outliers in the "Political and Legislative Actions" dimension.

- **Preventive, mitigative, and protective measures**: Evaluated through three dichotomous or multiple-choice response questions, forming a "Measures" dimension (11 variables, α = .716).

Question	Variables	Answer
26. Indicate the measures you have taken to protect yourself or your family from radon	26_1 Increase ventilation of the crawl space	Multiple choice
	26_2 Install a mechanical radon evacuation system in the basement or under solid floors	
	26_3 Prevent radon from seeping from the basement into living areas	
	26_4 Seal floors and walls	
	26_5 Improve building ventilation, especially in the context of energy savings	
	~~26_6 None~~	
	~~26_7 No answer~~	
27. Have you taken any action related to radon (measuring, remediation, informing) or do you know someone who has?	27 Actions taken against radon	Yes; No
28. Indicate which of the following statements are true and which are false	28_1 If I had a radon problem, it would be expensive to fix	True; False; Don't know/No answer
	28_2 I don't know how to test for radon in my home	
	28_3 I don't know where to buy a radon test kit	
	28_4 Radon test results are unreliable	
	~~28_5 I don't have time to test for radon in my home~~	
	28_6 If I tested for radon in my home and the results showed unacceptable levels, I wouldn't know how to find a contractor with radon experience to fix the problem	

Note: Struck-through variables were excluded for being outliers in the "Measures" dimension.

- **Information habits and communication actions on radon**: Assessed via Likert scale (1–7), multiple-choice, and dichotomous response questions.

Question	Variables	Answer
10a. How would you rate your interest in staying informed about what is happening around you and in the world?	10a Interest in staying informed	1 (Not interested at all) - 7 (Extremely interested)
10b. Do you actively avoid the news?	10b News avoidance	1 (Strongly disagree) - 7 (Strongly agree)
10c. Which of the following media did you consult for information during the past week?	10c Media channels	Print press; Digital media; Television; Radio; Twitter; Facebook; Instagram; TikTok; Twitch; YouTube; Signal; Telegram; WhatsApp; Zalo; Others (specify)
10d. How frequently do you consult the following media for information?	10d_1 Print press	Several times a day; Daily; Several times a week; Weekly; Several times a month; Monthly; Occasionally; Never
	10d_2 Digital media	
	10d_3 Television	
	10d_4 Radio	
	10d_5 Social media	
	10d_6 Instant messaging apps	
11a. Through which of the following channels have you received information about radon at any point?	11a Media channels on radon	Print press; Digital media; Television; Radio; Twitter; Facebook; Instagram; TikTok; Twitch; YouTube; Signal; Telegram; WhatsApp; Zalo; Others (specify); None
11b. Through which of the following channels have you received information about radon in the past week?	11b Media channels on radon (past week)	
12. Could you specify the name of the media where you found information about radon?	12 Media outlets	Open-ended
13. Who provided the information about radon content?	13_1 Government	Multiple choice
	13_2 NGO	
	13_3 Political party	
	13_4 Nuclear Safety Council	
	13_5 Don't remember	
	13_6 Others (please specify below)	
14. How often do you post messages on social media?	14 Social media use	Once a day; Several times a day; About once a week; Several times a week; Never
15. How often do you post messages on social media about risk situations?	15 Social media use (risk)	Never; Once a day; Several times a day; About once a week; Several times a week; Regularly

**Question**	**Variables**	**Answer**
29. Indicate whether you have received information about radon from any of the following institutions or groups	29_1 School	Yes; No
	29_2 University	
	29_3 Workplace	
	29_4 Media	
	29_5 National government	
	29_6 Regional government	
	29_7 Local government	
	29_8 European Union	
	29_9 Scientific organizations	
	29_10 Nuclear Safety Council	
	29_11 Through friends and family	
	29_12 Others (please specify)	
	29_13 None	
	29_14 No answer	
30. Indicate your level of trust in the following institutions or groups	30_1 School	1 (No trust at all) - 7 (Very high trust)
	30_2 University	
	30_3 Workplace	
	30_4 Media	
	30_5 National government	
	30_6 Regional government	
	30_7 Local government	
	30_8 European Union	
	30_9 Scientific organizations	
	30_10 Nuclear Safety Council	
	30_11 Friends and family	
31. What types of materials do you know of for communicating messages about radon? (e.g., brochures, posters, videos, CDs, DVDs, promotional products, exhibitions, talks, etc.)	31 Communication materials	
32. Have specific radon risk communication activities been carried out in your country?	32 Radon communication activities	Yes; No; Don't know/No answer
33. What would be the best channels, formats, and materials for this type of communication?	33_1 Brochures	Multiple choice
	33_2 Posters	
	33_3 Videos	
	33_4 CD/DVD	
	33_5 Talks	
	33_6 Media	
	33_7 Social media	
	33_8 Others	
	33_9 Don’t know	
